# Research on core competencies of university English teachers and pathway optimization based on fsQCA

**DOI:** 10.3389/fpsyg.2026.1815384

**Published:** 2026-07-02

**Authors:** Taocheng Jiang, Feng Wang, Runzi Xiao

**Affiliations:** 1 School of Foreign Languages, Xiangnan University, Chenzhou, China; 2 School of Physical Education, Xiangnan University, Chenzhou, China

**Keywords:** factor analysis, fuzzy-set qualitative comparative (fsQCA), occupational honor, teacher professionalism, university English teachers

## Abstract

**Introduction:**

This study aims to examine how individual, organizational, and social resources jointly shape the professional core competencies of college English teachers from a configurational perspective. It integrates Expectancy-Value Theory, the Job Demands-Resources Model, and Social Capital Theory to explain the formation of professional core competencies.

**Methods:**

A questionnaire on the influencing factors of professional core competencies among college English teachers was developed and validated. Data were collected from 561 college English teachers across 17 universities in East, Central, and Southwest China through stratified random sampling combined with snowball sampling. Fuzzy-set Qualitative Comparative Analysis was employed to explore the configuration effects and pathways of these influencing factors.

**Results:**

The findings show that external training support and self-efficacy-professional commitment serve as recurrent core conditions across sufficient configurations rather than as single necessary conditions. Other factors demonstrate resource complementarity effects, forming four effective configuration pathways: comprehensive empowerment, collaboration-policy dual-core, policy-efficiency support, and incentive-deficit compensation. These pathways indicate that professional competency formation is characterized by equifinality, resource complementarity, and functional substitution.

**Discussion:**

The high-level development of professional core competencies among college English teachers results from the synergistic coupling of personal resources, organizational resources, and social capital. The findings suggest that higher education administrators should optimize teacher training systems, improve incentive mechanisms, build collaborative platforms, and provide time management support. Theoretically, this study extends research on teacher professional development by demonstrating the value of configurational causality in understanding professional core competencies.

## 1 Introduction

The professional development of university teachers has become a central concern in higher education research because teaching quality, student learning outcomes, and institutional competitiveness increasingly depend on teachers’ capacity to integrate disciplinary knowledge, pedagogical expertise, digital competence, research literacy, and continuous learning. In university English education, this issue is particularly important. University English teachers are expected not only to deliver language instruction, but also to support intercultural communication, curriculum innovation, digital learning, and students’ broader academic development. Therefore, enhancing the professional competence of university English teachers has long been one of the key measures to improve the quality of English talent cultivation in higher education (Zhang, 2019).

Teacher professional competence is a multidimensional concept. The latest research, the “Standards for Professional Competence of University Foreign Language Teachers,” summarises the competencies that university foreign language teachers should possess into six dimensions: educational competence, disciplinary competence, teaching competence, research competence, digital competence and learning competence (Zhang and Xu, 2024). These dimensions are interrelated yet each emphasises different aspects, collectively forming a comprehensive framework for evaluating the professional level of foreign language teachers. Meanwhile, the international higher education community has also explored the common characteristics of “teaching excellence,” generally recognising that excellent teachers should have good teacher-student interaction skills, promote effective active learning, set high yet achievable expectations, respect student diversity, possess effective communication abilities and invest in the continuous improvement of teaching (Hammer et al., 2010). In this study, professional core competencies refer to the integrated psychological, pedagogical, disciplinary, digital, and developmental capacities that enable university English teachers to achieve sustained professional growth and teaching excellence. Teaching excellence is regarded as the visible performance outcome of these competencies, whereas Objective Honor Attainment (OHA) is used as an institutionalized indicator of objective career recognition. This operationalization is consistent with the distinction between subjective and objective career success, in which objective career success is commonly represented by externally verifiable indicators such as rank, title, awards, and recognized achievements (Arthur et al., 2005). OHA does not represent the whole meaning of professional competence; rather, it provides a measurable outcome through which teachers’ externally recognized professional success can be empirically examined.

The relationship between teachers’ professional literacy and teaching honours has been receiving increasing attention both domestically and internationally. In China, in recent years, scholars have focused on topics such as information literacy (Wang and Tang, 2022), integration of ideological and political education into the curriculum (Sun, 2022), and the status and enhancement of professional literacy (He, 2025). Studies on teaching awards and professional recognition further suggest that institutional rewards alone do not automatically improve teaching quality; instead, teachers’ intrinsic motivation, professional confidence, supportive environments, and collaborative cultures are crucial for transforming recognition into sustained professional development (Gibbs and Coffey, 2004; Kreber and Brook, 2001; Menges, 1996). Thus, the development of university English teachers’ professional core competencies should be understood as a process jointly shaped by individual motivation, organizational resources, and social capital.

Despite these contributions, three limitations remain. First, professional competence, teaching excellence, and occupational honor have often been examined as relatively separate topics, leading to insufficient conceptual integration between competence formation and objective recognition. Second, most empirical studies have relied on linear models that estimate the independent net effects of single variables, such as self-efficacy, organizational support, training participation, or collaborative culture. Such approaches are useful but limited because they cannot adequately explain whether different combinations of resources may produce the same outcome. Third, previous research has paid insufficient attention to equifinality and causal asymmetry in teacher professional development. The conditions associated with high OHA may not simply be the opposite of those associated with low OHA, and different institutional contexts may support teacher success through different but functionally equivalent configurations. Therefore, it is necessary to move beyond single-factor and linear explanations toward a configurational understanding of professional core competency formation.

To address these limitations, this study integrates Expectancy–Value Theory, the Job Demands–Resources Model, and Social Capital Theory into a unified explanatory framework. Expectancy–Value Theory explains why teachers are motivated to pursue professional growth and teaching honor: teachers are more likely to invest sustained effort when they believe success is attainable and professionally valuable (Eccles and Wigfield, 2002). The Job Demands–Resources Model explains how personal and organizational resources help teachers cope with job demands and transform professional effort into performance outcomes (Bakker and Demerouti, 2017). Social Capital Theory further explains how relational and network-based resources, especially peer collaboration and external training networks, expand teachers’ access to information, recognition opportunities, and professional communities (Putnam, 2000). Together, these theories suggest that high-level professional competence and OHA are unlikely to result from one dominant factor; instead, they are more plausibly produced by the interaction and alignment of motivational, organizational, and relational resources.

Based on prior literature and the integrated theoretical framework, the 60 questionnaire items were initially categorised into seven potential factors: Teacher Self-Efficacy and Career Commitment (SEC), Time–Stress Management (TSM), Organisational Policy Support (OPS), External Training Support (ETS), Peer Collaboration Culture (PCC), Incentives and Promotion Mechanisms (IPM), and School Career Development Environment (ICDC). These factors encompass individual resources, organisational resources, and social capital, providing a comprehensive perspective for explaining teachers’ OHA (Wang and Sun, 2012; Ebner and Paul, 2023). Rather than assuming that these factors influence professional success in an additive and symmetric manner, this study examines how they combine into sufficient configurations. Fuzzy-set qualitative comparative analysis (fsQCA) is therefore methodologically necessary because the research problem involves conjunctural causation, resource complementarity, functional substitution, and multiple sufficient pathways (Ragin, 2008; Fiss, 2011).

Accordingly, this study addresses three substantive research questions: first, what individual, organizational, and social-capital-related factors constitute the key resource structure associated with university English teachers’ professional core competencies and objective honor attainment? Second, how do these factors combine to generate high-level OHA among university English teachers? Third, what forms of resource complementarity, functional substitution, and equifinal pathways characterize the professional success of university English teachers across different institutional contexts?

This study contributes to the literature in three ways. First, it offers an integrated conceptual model linking professional core competencies, teaching excellence, and objective honor attainment in the context of university English teachers. Second, it extends educational psychology research on teacher professional development by explaining how motivation, resources, and relational networks jointly shape professional success. Third, by adopting a configurational perspective, it demonstrates that teacher professional success is not generated through a single linear pathway, but through multiple, causally asymmetric, and functionally equivalent configurations.

## 2 Research methods

### 2.1 Research design

This study adopted a sequential multi-method quantitative design. All three analytical procedures—exploratory factor analysis (EFA), confirmatory factor analysis (CFA), and fuzzy-set qualitative comparative analysis (fsQCA)—belong to the quantitative domain, but they served different methodological purposes. First, exploratory factor analysis (EFA) was used to extract latent structures from 60 questionnaire items. Then, confirmatory factor analysis (CFA) was employed to test the fit of the seven-factor measurement model. Finally, fuzzy-set qualitative comparative analysis (fsQCA) was used to explore the sufficient and necessary conditions of different resource–capability configurations for Chinese university English teachers’ objective honour attainment (OHA). Thus, EFA and CFA were used as measurement validation procedures, whereas fsQCA was used to examine configurational causality, equifinality, and causal asymmetry in the formation of professional core competencies and objective professional recognition.

### 2.2 Participants and sampling

The study sample consisted of English teachers from 17 universities in three major regions: East China, Central China and Southwest China. To enhance regional and institutional diversity, the study first adopted a stratified recruitment strategy based on geographical region and type of institution. Within each stratum, university English teachers were invited through teaching departments and professional networks. Because university English teachers are a relatively dispersed professional group and access to institutional sampling frames was limited, snowball recruitment was used only as a supplementary strategy to reach eligible participants within the same professional population. Therefore, the sampling procedure should be understood as a stratified purposive recruitment process supplemented by snowball sampling, rather than as a purely probability-based random sample.

A total of 610 questionnaires were returned. After data screening, including checks for incomplete responses, patterned responses, and multivariate outliers based on Mahalanobis D^2^, 561 valid responses were retained, with a sample-to-item ratio of approximately 8:1, meeting the minimum requirements for EFA and CFA (Hair et al., 2019). All participants were university English teachers who were currently engaged in English language teaching, curriculum development, or related professional work in higher education institutions.

### 2.3 Measures and construct operationalization

Measurement tools consisted of seven potential dimensions and one outcome variable. The questionnaire initially included 60 items covering these seven theoretically derived constructs (Table 1). Teacher self-efficacy and career commitment (SEC), time–pressure management ability (TSM), organisational policy support (OPS), external training support (ETS), peer collaboration culture (PCC), incentive and promotion mechanisms (IPM), and school career development environment (ICDC) are all scored using a seven-point Likert scale. These seven conditions were selected based on the integrated theoretical framework of Expectancy–Value Theory, the Job Demands–Resources Model, and Social Capital Theory. Table 1 presents the core meaning and an example item for each construct. The table is intended to describe the measurement design, while the final item structure determined through EFA and CFA is reported in the Results section. Specifically, SEC reflects teachers’ motivational belief and professional commitment; TSM reflects individual capacity to manage job demands; OPS, ETS, IPM, and ICDC reflect organizational and institutional resources; and PCC reflects relational and social-capital resources.

**Table 1:** Measurement constructs, core meanings, and example items.

Construct	Core meaning	Example item
SEC	Self-efficacy and professional commitment	“I am confident that I can effectively complete my teaching tasks.”
TSM	Time-stress management	“I can balance teaching tasks with personal life demands.”
OPS	Organizational policy support	“School policies support my professional development.”
ETS	External training support	“The school funds teachers to attend professional development-related training.”
PCC	Peer collaboration culture	“I can participate in projects or research with colleagues.”
IPM	Incentive and promotion mechanism	“The school’s incentive measures are attractive.”
ICDC	Institutional career development environment	“The school values teacher development and provides support and resources.”

The outcome variable OHA represents objective career success by a six-level ordinal scoring from 0 to 5 based on the highest award level attained by teachers in the past 5 years: 0 = no award, 1 = school-level award, 2 = county/district-level award, 3 = city-level award, 4 = provincial-level award, and 5 = national-level award. OHA was treated as an ordinal indicator of objective professional recognition. It does not represent the whole construct of professional core competence; rather, it serves as an institutionalized and externally observable outcome associated with teachers’ professional visibility, teaching excellence, and recognized career achievement. This operationalization is consistent with the distinction between subjective career success and objective career success, where objective success is usually captured through externally verifiable indicators such as rank, title, award, or recognized achievement (Arthur et al., 2005).

Scale items are derived from established tools (Rahimi, 2024) and revised with semi-structured interviews, then reviewed in two rounds using the Delphi method by three measurement and language PhDs to ensure content validity. Items were adapted to the context of university English teaching and were reviewed for conceptual clarity, linguistic appropriateness, and relevance to teacher professional development.

### 2.4 Data collection and ethics

Online questionnaires were distributed through Wenjuanxing after informed consent was obtained. Before completing the questionnaire, participants were informed of the research purpose, voluntary nature of participation, approximate completion time, confidentiality procedures, and their right to withdraw at any time without penalty. No personally identifiable information was collected. The study involving human participants was reviewed and approved by the Ethics Committee of Xiangnan University (Approval No. K/KYX2025-045-01; Approval Date: 13 November 2025). All data were anonymized, stored securely, and used only for academic research purposes. The study was conducted in accordance with the ethical standards for research involving human participants and the requirements of the participating institutions.

### 2.5 Data screening and missing data treatment

Before formal analysis, the dataset was screened for missing values, careless or patterned responses, normality, and multivariate outliers. Cases with substantial missing information, invalid response patterns, or multivariate outliers identified by Mahalanobis D^2^ were removed. After this screening procedure, 561 valid responses were retained from the 610 returned questionnaires. The retained dataset contained complete information for the variables used in the subsequent analyses; therefore, no missing-value imputation was applied.

Normality diagnostics were conducted using skewness and kurtosis statistics to assess whether the observed variables showed serious departures from distributional assumptions. Because EFA, CFA, and fsQCA have different analytical assumptions, these diagnostics were used mainly to determine whether additional robustness checks or cautious interpretation were required. Detailed screening results are reported in the Results section rather than in the Methods section.

### 2.6 Measurement validation procedures

Internal consistency was evaluated using Cronbach’s *α* and item–total correlation (CITC), with thresholds set at 0.70 and 0.40, respectively (Nunnally and Bernstein, 1994). Data suitability was determined through the Kaiser–Meyer–Olkin measure (Kaiser, 1974) and Bartlett’s test of sphericity. Only the criteria and procedures are described in this section; specific Cronbach’s α, KMO, and Bartlett’s test values are reported in the Results section to maintain a clear separation between methods and findings.

Factor extraction was conducted using principal axis factoring with parallel analysis to assist in determining the number of factors. An oblique rotation method was preferred because the latent dimensions of teacher professional development were theoretically expected to be correlated. Items were retained or removed based on factor loading, cross-loading, conceptual relevance, and the interpretability of the factor structure.

After EFA, CFA was conducted to examine the stability of the measurement model. Model fit was evaluated using commonly reported indices, including χ^2^/df, CFI, TLI, RMSEA, SRMR, AIC, and BIC (Hu and Bentler, 1999). Composite reliability, average variance extracted, and discriminant validity were then examined to assess the reliability and validity of the final measurement model.

## 3 fsQCA calibration and configuration analysis

After validating the measurement model, factor scores were used for fsQCA. The seven condition variables were calibrated using the direct calibration method. For Likert-scale condition variables, the 90th percentile, 50th percentile, and 10th percentile were used as anchors for full membership, crossover point, and full non-membership, respectively. This percentile-based calibration was used because the study focused on relative membership in resource conditions within the sample of university English teachers.

For the outcome variable OHA, calibration was based on both theoretical meaning and the ordinal structure of the award hierarchy. Teachers with provincial-level or national-level awards were treated as having high membership in the set of high OHA; teachers with no awards were treated as having low membership; and intermediate award levels were positioned between these two anchors. This approach avoids treating all award levels as interval-scale values and better reflects the ordinal and institutional nature of professional honor attainment.

The fsQCA analysis proceeded in three steps. First, necessary condition analysis was conducted, with a conventional consistency threshold of 0.90 for judging whether a single condition could be considered necessary for high OHA. Second, a truth table was constructed for sufficiency analysis. Considering the sample size of 561 and the number of causal conditions, the frequency threshold was set at 2. This threshold was used to avoid retaining configurations represented by only one case while preserving sufficient empirical diversity for configurational comparison. Third, the truth table was minimized to obtain complex, parsimonious, and intermediate solutions. Consistency and coverage were reported to evaluate the empirical relevance and explanatory strength of each configuration.

Frequency = 1 was not used as the main analytical threshold. In interpreting the sufficiency results, configurations with consistency values above the conventional 0.80 benchmark were treated as robust sufficient configurations. Configurations below this benchmark were interpreted more cautiously as supplementary patterns rather than as robust sufficient pathways. This distinction was applied to ensure that the interpretation of the fsQCA results remained consistent with both methodological standards and the empirical strength of each configuration (Fiss, 2011).

Core and peripheral conditions were identified by comparing the parsimonious and intermediate solutions. Conditions appearing in both solutions were treated as core conditions, whereas conditions appearing only in the intermediate solution were treated as peripheral conditions.

## 4 Results

### 4.1 Descriptive statistics and data screening

A total of 610 questionnaires were returned, and 561 valid responses were retained after excluding incomplete responses, patterned responses, and multivariate outliers based on Mahalanobis D^2^. The final sample consisted of university English teachers from 17 universities in East China, Central China and Southwest China. As shown in Table 2, the sample covered teachers with different demographic, professional, and institutional backgrounds, which provided an adequate empirical basis for subsequent measurement validation and configurational analysis.

**Table 2:** Sample characteristics and outcome distribution.

Variable	Descriptive result
Returned questionnaires	610
Valid responses	561
Source institutions	17 universities
Regions	East China, Central China, and Southwest China
Female teachers	66.50%
Main age group	36–40 years
Teaching experience	M = 16.76, SD = 10.88, Median = 18
Main professional title	Lecturer
Teacher education background	55.10%
Main institution type	Regular undergraduate institutions
Main city level	Second-tier cities
OHA	M = 0.91, SD = 1.38, Median = 0
No teaching-related honor	73.60%
School-level or higher honor	26.40%
Highest observed honor level	National level

OHA, Objective Honor Attainment. OHA was coded from 0 to 5, where 0 = no award, 1 = school-level award, 2 = county/district-level award, 3 = city-level award, 4 = provincial-level award, and 5 = national-level award.

The proportion of female teachers in the study sample is relatively high (66.5%), with ages concentrated in the 36–40 range (M = 4.29, corresponding to the fifth level), reflecting a predominance of mid-career teachers. The average teaching experience of respondents is approximately 16.76 years (SD = 10.88), with a median of 18 years, indicating generally substantial teaching experience. The distribution of professional titles is mainly intermediate (lecturer) (Md = 3), and more than half of the respondents have a background in teacher education (55.1%). In terms of the types of schools taught, teachers in general undergraduate institutions predominate (M = 1.96), with many schools located in second-tier cities (M = 2.01).

Regarding objective professional honours, the average honour level is 0.91 (SD = 1.38), with a median of 0, indicating that 73.6% have not received any awards; however, 26.4% of teachers have received school-level or higher honours, with the highest reaching the national level (level 5). This uneven distribution is theoretically meaningful because high-level professional recognition is selective and institutionally stratified. Therefore, OHA was treated as an ordinal indicator of objective professional recognition rather than as a normally distributed continuous outcome.

Data screening further indicated that the retained dataset was suitable for subsequent analysis. Missing values were examined before formal analysis, and remaining missing responses were handled using the expectation–maximization procedure described in the Methods section. Normality and homogeneity diagnostics were conducted to determine whether robust estimation or additional sensitivity checks were required. Overall, the retained sample and screened dataset provided a reliable basis for EFA, CFA, and fsQCA.

### 4.2 Measurement model validation

Before conducting the configurational analysis, the measurement model was validated to ensure that the seven theoretical constructs could be measured reliably and distinctly. To examine the internal consistency of the scale, this study calculated the overall Cronbach’s *α* coefficient for the 60 items across seven latent dimensions, which was 0.963, far exceeding the recommended threshold of 0.70. Further item-total correlation analysis showed that the corrected item-total correlations were above the minimum criterion of 0.40. These results indicate that the questionnaire had adequate internal consistency for subsequent factor analysis.

To confirm the suitability of the data for factor extraction, the Kaiser–Meyer–Olkin measure and Bartlett’s test of sphericity were conducted. The KMO value was 0.958, and Bartlett’s test of sphericity was significant, χ^2^(1770) = 15,205.192, *p* < 0.001. These results confirmed that the correlation matrix was appropriate for exploratory factor analysis.

Exploratory factor analysis was then conducted to examine the latent structure of the initial item pool. Rather than reporting the full item-by-item elimination process in the main text, only the main results are presented here to maintain concision. After removing weakly performing or conceptually ambiguous items, a seven-factor structure was retained. The seven factors corresponded to Teacher Self-Efficacy and Career Commitment (SEC), Time–Stress Management (TSM), Organisational Policy Support (OPS), External Training Support (ETS), Peer Collaboration Culture (PCC), Incentives and Promotion Mechanisms (IPM), and School Career Development Environment (ICDC). The final EFA solution explained 53.34% of the total variance, indicating an acceptable multidimensional structure for applied educational and psychological research.

Confirmatory factor analysis was further conducted to examine the stability of the seven-factor measurement model. The initial 42-item model was compared with a more parsimonious 30-item model after removing items with weak loadings, conceptual ambiguity, or local misfit. Item refinement was guided by both statistical criteria and theoretical relevance, with the aim of achieving an acceptable balance between model fit, construct clarity, and model parsimony. As shown in Table 3, the revised 30-item model showed acceptable fit: χ^2^/df = 2.11, GFI = 0.91, CFI = 0.935, TLI = 0.928, RMSEA = 0.045, and SRMR = 0.053. Compared with the initial model, the revised model also showed lower AIC and BIC values, supporting its greater parsimony. Therefore, the 30-item seven-factor model was retained for subsequent analysis.

**Table 3:** Summary of measurement model validation.

Validation aspect	Indicator	Result	Interpretation
Internal consistency	Overall Cronbach’s α	0.963	Excellent
Item consistency	CITC	> 0.40	Acceptable
Sampling adequacy	KMO	0.958	Excellent
Factorability	Bartlett’s test	χ^2^(1770) = 15,205.192, *p* < 0.001	Significant
EFA solution	Cumulative variance explained	53.34%	Acceptable
CFA fit	χ^2^/df	2.11	Acceptable
CFA fit	GFI	0.91	Acceptable
CFA fit	CFI	0.935	Good
CFA fit	TLI	0.928	Good
CFA fit	RMSEA	0.045	Good
CFA fit	SRMR	0.053	Acceptable
Model parsimony	AIC/BIC	131.00/421.09	Lower than initial model

EFA, exploratory factor analysis; CFA, confirmatory factor analysis; CITC, corrected item-total correlation.

The composite reliability of the seven latent variables ranged from 0.753 to 0.829, all exceeding the recommended threshold of 0.70. The average variance extracted ranged from 0.433 to 0.538. Although several AVE values were slightly below the ideal threshold of 0.50, this does not necessarily invalidate convergent validity. According to Fornell and Larcker (1981), when AVE is slightly lower than 0.50 but composite reliability exceeds 0.60, convergent validity may still be considered acceptable. In this study, all CR values exceeded 0.70, indicating that the constructs retained adequate reliability despite borderline AVE values. The square roots of AVE were also examined in relation to inter-construct correlations. The results generally supported discriminant validity, although relatively high correlations between SEC and TSM, and between ICDC and IPM, suggest that these constructs are theoretically related but not empirically redundant (Table 4).

**Table 4:** Reliability and convergent validity of the seven constructs.

Construct	CR	AVE	√AVE	Interpretation
SEC	0.814	0.467	0.683	Acceptable
TSM	0.788	0.482	0.694	Acceptable
OPS	0.769	0.455	0.674	Borderline AVE; acceptable CR
ETS	0.792	0.489	0.699	Borderline AVE; acceptable CR
PCC	0.823	0.538	0.733	Good
IPM	0.753	0.433	0.658	Borderline AVE; acceptable CR
ICDC	0.829	0.493	0.702	Borderline AVE; acceptable CR

CR, composite reliability; AVE, average variance extracted. AVE values slightly below 0.50 were interpreted together with CR values following Fornell and Larcker’s criterion.

Overall, the EFA and CFA results support the use of SEC, TSM, OPS, ETS, PCC, IPM, and ICDC as empirically distinguishable conditions in the subsequent fsQCA. This step is methodologically important because fsQCA requires conceptually meaningful and sufficiently reliable conditions before they can be calibrated and combined into causal configurations. In this study, measurement validation therefore served as the empirical bridge between the theoretical framework and the later configurational analysis.

## 5 fsQCA calibration and necessary condition analysis

After the measurement model was validated, the seven calibrated constructs were used as condition variables in fsQCA. To maintain consistency between quantitative measurement and fuzzy-set logic, all seven condition variables—SEC, TSM, OPS, ETS, PCC, IPM, and ICDC—were calibrated using the direct calibration method. The 90th, 50th, and 10th percentiles of each condition variable were used as anchors for full membership, crossover point, and full non-membership, respectively. This percentile-based calibration was adopted because the study examined relative membership in different resource conditions within the sample of university English teachers.

The outcome variable, Objective Honor Attainment (OHA), was calibrated according to both its ordinal structure and theoretical meaning. Teachers who obtained provincial-level or national-level teaching-related honors were treated as having high membership in the set of high OHA, teachers without any teaching-related honor were treated as having low membership, and intermediate honor levels were calibrated between these two anchors. This approach avoids treating the award hierarchy as a purely interval-scale variable and better reflects the institutional stratification of professional recognition.

Necessary condition analysis was conducted before sufficiency analysis to determine whether any single condition was indispensable for high OHA. Following the conventional fsQCA criterion, a condition was considered necessary only when its consistency reached or exceeded 0.90. As shown in Table 5, none of the seven positive conditions reached this threshold. Self-efficacy and professional commitment (SEC) showed the highest consistency value 0.852, followed by TSM 0.789 and ETS 0.788, but all remained below the necessity threshold. The consistency values of the negated conditions were also low, ranging from 0.214 to 0.333. Therefore, no single condition or its absence can be regarded as a necessary condition for high OHA.

**Table 5:** Necessary condition analysis for high OHA.

Conditions	Consistency	Coverage
External Training Support (ETS)	0.788285	0.635173
~External Training Support (ETS)	0.276205	0.239542
Self-Efficacy - Professional Commitment (SEC)	0.851650	0.693360
~Self-Efficacy - Professional Commitment (SEC)	0.214154	0.183694
Incentive - Promotion Mechanism (IPM)	0.760734	0.603694
~Incentive - Promotion Mechanism (IPM)	0.303759	0.267869
Peer Collaboration Culture (PCC)	0.761468	0.627460
~Peer Collaboration Culture (PCC)	0.304501	0.257933
Overall Career Development Environment (ICDC)	0.744440	0.619419
~Overall Career Development Environment (ICDC)	0.320505	0.268818
Time - Stress Management Ability (TSM)	0.789147	0.636832
~Time - Stress Management Ability (TSM)	0.276077	0.239041
Policy Support (OPS)	0.732764	0.624222
~Policy Support (OPS)	0.332511	0.272499

A condition is generally considered necessary when consistency ≥ 0.90. None of the positive conditions reached this threshold. The consistency values for the negated conditions ranged from 0.214 to 0.333 and therefore also failed to meet the necessity criterion.

This finding provides an empirical basis for conducting sufficiency analysis. It suggests that high OHA is not generated by any isolated resource or capability, but is more likely to emerge from specific combinations of motivational, organizational, and social-capital-related conditions.

### 5.1 Sufficient configurations for high OHA

After the necessary condition analysis, sufficiency analysis was conducted to identify combinations of conditions associated with high OHA. The truth table was minimized using a frequency threshold of 2. Given the sample size of 561, this threshold avoids retaining configurations represented by only one case while preserving sufficient empirical diversity. The overall solution consistency was 0.841, and the overall solution coverage was 0.656, indicating that the identified configurations explained approximately 65.6% of high-OHA cases.

As shown in Table 6, three configurations showed strong consistency values above 0.80, while the fourth configuration showed a lower consistency value of 0.723. Therefore, P1–P3 are interpreted as robust sufficient configurations, whereas P4 is retained as a supplementary compensatory pattern and should be interpreted with caution. Across the configurations, external training support (ETS) and self-efficacy and professional commitment (SEC) appeared repeatedly as core conditions. However, because neither ETS nor SEC reached the necessity threshold in Table 5, they should not be described as necessary conditions. Instead, they are more accurately interpreted as recurrent core conditions within sufficient configurations.

**Table 6:** Configurations for high objective honor attainment.

Conditions	P1	P2	P3	P4 Supplementary
ETS	●	●	●	●
SEC	●	●	●	●
IPM	•	•	•	⊙
PCC	•	•		•
ICDC	•	•	•	⊙
TSM	•		•	•
OPS		•	•	•
Consistency	0.884	0.88	0.88	0.723
Raw coverage	0.617	0.616	0.608	0.23
Unique coverage	0.018	0.017	0.009	0.013

● = core condition present; • = peripheral condition present; ⊙ = peripheral condition absent; blank = condition irrelevant or “do not care.” The symbols distinguish recurrent central conditions from context-dependent accompanying conditions. Because the current analysis is based primarily on the intermediate solution, the core/peripheral distinction should be interpreted cautiously.

P1, the comprehensive empowerment configuration, represents a resource-enrichment pathway. In this configuration, ETS and SEC are accompanied by incentive and promotion mechanisms, peer collaboration culture, institutional career development environment, and time–stress management ability. This indicates that high OHA is most strongly supported when teachers’ professional motivation, external training opportunities, institutional incentives, collaborative support, and personal resource-management ability are simultaneously present. This pathway has the highest consistency 0.884 and raw coverage 0.617, suggesting that it has strong explanatory relevance among high-OHA cases.

P2, the collaboration–policy support configuration, also reflects a resource-enrichment logic but with a stronger emphasis on institutional and relational support. In this pathway, ETS and SEC are combined with incentive mechanisms, peer collaboration culture, institutional career development environment, and organizational policy support, while TSM is not a decisive condition. This suggests that clear policy support and collaborative culture may help compensate for differences in individual time–stress management when other developmental resources are available.

P3, the policy–efficiency support configuration, reflects a more compensatory pattern. In this configuration, ETS and SEC are combined with incentive mechanisms, institutional career development environment, time–stress management ability, and organizational policy support, while peer collaboration culture is not a decisive condition. This indicates that when peer collaboration is limited or less salient, clear policy support and teachers’ own time–stress management ability may still support high OHA.

P4, the incentive-deficit compensation configuration, should be interpreted more cautiously because its consistency value is lower than the conventional 0.80 threshold. This configuration combines ETS, SEC, peer collaboration culture, time–stress management ability, and organizational policy support, while incentive and promotion mechanisms and institutional career development environment are absent. Although it is weaker than P1–P3, P4 suggests a supplementary compensatory logic: in relatively resource-constrained contexts, the absence of formal incentives and a strong career development environment may be partly offset by training access, peer collaboration, individual regulation, and policy support.

Overall, the sufficiency analysis indicates that high OHA is produced through multiple pathways rather than a single optimal route. The first two configurations mainly reflect resource-enrichment mechanisms, whereas the latter two configurations suggest resource-compensation mechanisms. This result provides the empirical basis for the discussion section, where these configurations are further interpreted in relation to Expectancy–Value Theory, the JD-R model, and Social Capital Theory.

## 6 Discussion

### 6.1 Measurement validation and the legitimacy of configurational analysis

The measurement validation results provide an important basis for interpreting the configurational findings. In this study, EFA and CFA were not used as independent research purposes, but as necessary procedures to examine whether the seven theoretically derived constructs could be measured reliably and distinctly. The acceptable model fit, composite reliability, and discriminant validity indicate that SEC, TSM, OPS, ETS, PCC, IPM, and ICDC are related but distinguishable conditions. This distinction is important because fsQCA requires each condition to have sufficient conceptual clarity before it can be calibrated and combined into causal configurations.

Although several AVE values were slightly below the ideal threshold of 0.50, the constructs remained defensible because all CR values exceeded 0.70 and the discriminant validity results were generally acceptable. Following Fornell and Larcker (1981), convergent validity may still be supported when AVE is slightly below 0.50 but composite reliability is adequate. Therefore, the measurement model provides a reasonable empirical foundation for the subsequent fsQCA. In this sense, construct validation served as a bridge between the theoretical framework and the configurational explanation of high OHA.

### 6.2 Configurational model of university English teachers’ professional core competence development

The central finding of this study is that university English teachers’ professional core competence development is not a linear accumulation of individual ability, but a configurational process in which motivational activation, institutional resource alignment, and professional network support jointly generate externally recognized teaching excellence. This point is particularly important for university English teachers because their professional competence is inherently multidimensional. It involves not only general pedagogical competence, but also language pedagogy, intercultural communication, digital language teaching, curriculum innovation, and teaching-oriented research capacity.

**Figure 1:**
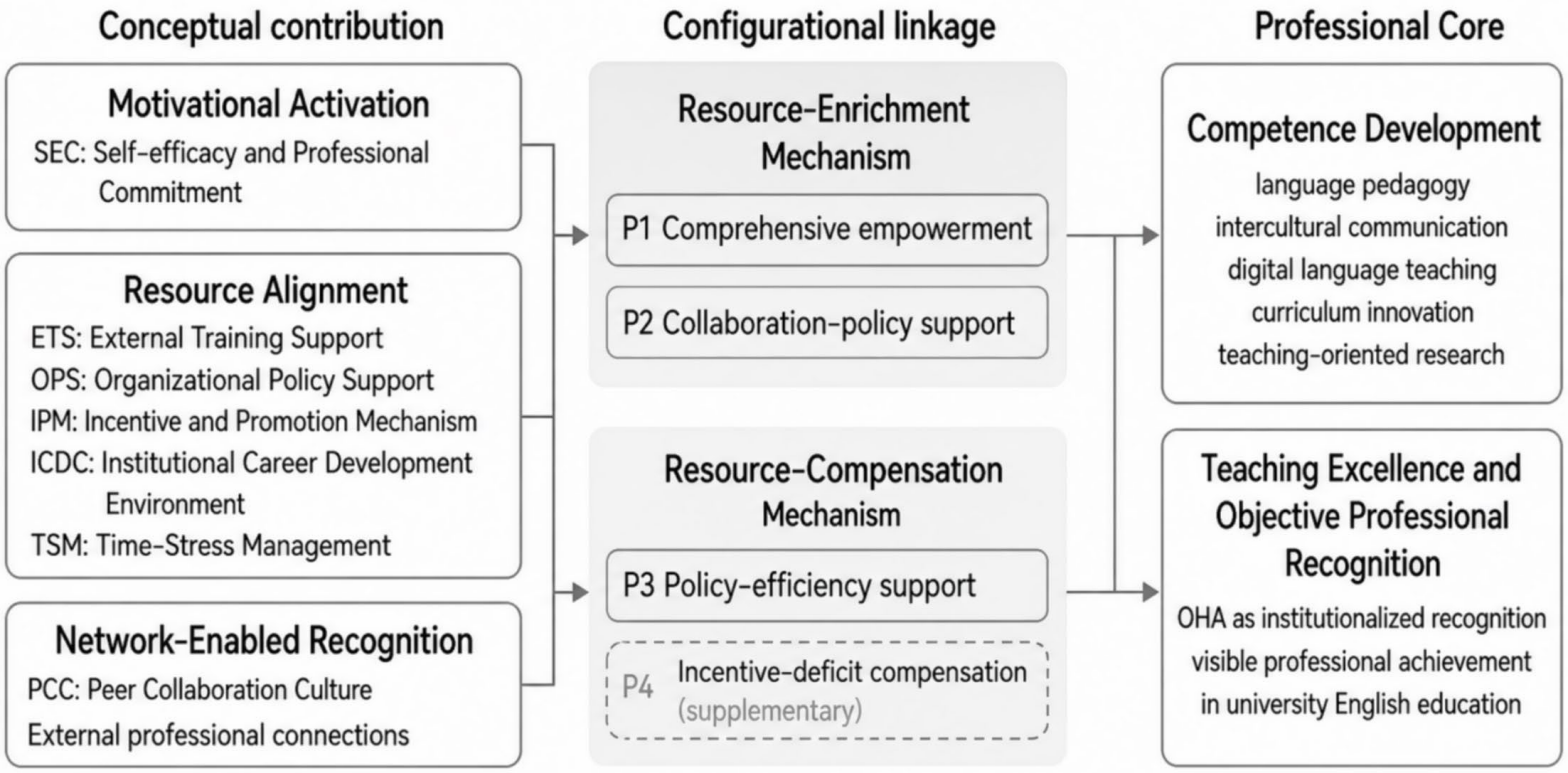
A configurational model of university English teachers’ professional core competence development. This model illustrates a configurational rather than linear logic. Resource-enrichment configurations reflect the joint reinforcement of motivation, training, institutional support, and collaboration, whereas resource-compensation configurations show how the absence of some resources may be partly offset by other motivational, organizational, or social-capital-related conditions. OHA is treated as an institutionalized indicator of externally recognized teaching excellence rather than as a complete measure of professional competence.

Based on the fsQCA results, this study proposes a configurational model of university English teachers’ professional core competence development. The model contains three interrelated mechanisms. The first is motivational activation, represented by teachers’ self-efficacy and professional commitment. Without a basic belief that professional effort is meaningful and attainable, teachers are less likely to sustain the long-term investment required for teaching innovation, curriculum reform, and professional recognition. The second is resource alignment, represented by external training support, organizational policy support, incentive mechanisms, career development environment, and time–stress management. These resources help teachers transform motivation into professional action. The third is network-enabled recognition, represented by peer collaboration and the external professional connections embedded in training and professional development opportunities. For university English teachers, professional visibility is often achieved through teaching-research groups, curriculum teams, teaching competitions, classroom observation, peer feedback, external training programs, and professional communities (Figure 1).

The four configurations should therefore not be understood merely as separate pathway labels. They reveal two broader mechanisms of professional core competence development. The first mechanism is resource enrichment, represented by the comprehensive empowerment configuration and the collaboration–policy support configuration. In these pathways, university English teachers obtain high OHA when professional motivation is supported by training opportunities, institutional incentives, collaborative culture, and career development platforms. This mechanism reflects a relatively resource-rich professional ecology in which individual effort and institutional support reinforce each other.

The second mechanism is resource compensation, represented by the policy–efficiency support configuration and the incentive-deficit compensation configuration. These pathways suggest that professional recognition can still emerge in less resource-rich contexts when some missing resources are compensated by others. For example, weaker peer collaboration may be offset by clear policy support and strong time–stress management, while insufficient formal incentives may be partly compensated by external training, peer collaboration, and policy support. This compensatory mechanism is especially relevant for local or teaching-oriented universities, where formal reward systems may be less developed but teachers can still achieve professional recognition through targeted support and collaborative professional practice.

Thus, the main conceptual contribution of this study is not simply the identification of four empirical configurations. Rather, the study shows that university English teachers’ professional core competence develops through different combinations of motivation, institutional resources, and professional networks. OHA, in this model, is not treated as a full substitute for competence itself, but as an institutionalized and externally visible outcome of professional growth, teaching excellence, and recognized contribution to university English education.

### 6.3 Theoretical implications for language teacher development

These findings offer theoretical implications for understanding language teacher development as a configurational rather than linear process. From the perspective of Expectancy–Value Theory, the recurrent role of SEC suggests that university English teachers’ self-efficacy and professional commitment provide an important motivational basis for professional development (Eccles and Wigfield, 2002). However, because SEC did not reach the necessity threshold, motivation should not be understood as an independent determinant of high OHA. Instead, its effect depends on whether it is combined with enabling conditions such as external training, institutional support, incentives, or peer collaboration. This finding refines EVT by showing that expectancy and value beliefs are activated through specific professional contexts rather than operating in isolation.

The findings also extend the Job Demands–Resources model. The JD-R model emphasizes that job resources can buffer demands and promote engagement and performance (Demerouti et al., 2001; Bakker and Demerouti, 2017; Chen and Chen, 2023). In this study, ETS appeared as a recurrent enabling condition, indicating that professional development resources are particularly important for university English teachers. Such resources connect teachers with updated language-teaching methodologies, digital learning tools, intercultural education resources, EAP/ESP curriculum reform, and broader professional communities. More importantly, ETS did not operate alone, but worked together with policy support, incentives, career development environment, collaboration, and time–stress management. This extends the JD-R model from a linear resource-effect explanation toward a configurational resource-alignment explanation.

The study further contributes to Social Capital Theory by clarifying the role of professional networks in language teacher development. Prior research shows that teachers’ professional learning is shaped by access to networks, shared knowledge, trust, and opportunities for interaction (Penuel et al., 2009; Demir, 2021; Coppe et al., 2022). In this study, peer collaboration culture reflects bonding social capital within institutions, whereas external training support may provide bridging social capital that links teachers to wider professional communities. However, collaboration was not always indispensable for high OHA. This finding qualifies existing research on teacher collaboration by suggesting that collaboration is one important resource within a broader configuration, rather than a universal prerequisite for professional recognition (Vangrieken et al., 2015).

Finally, the findings contribute to international research on teacher professional development by showing that widely recognized professional development features do not function as isolated best practices. Prior studies emphasize content focus, active learning, coherence, duration, collective participation, and opportunities for enactment in practice (Desimone, 2009; Avalos, 2011; Kennedy, 2016). This study adds that these features become effective through different combinations of motivation, institutional support, training access, and collaborative culture. This point is especially relevant for university English teachers, whose professional development increasingly involves digital language teaching, intercultural education, curriculum reform, and changing student learning needs. Recent research on EFL teachers’ professional and digital competence also emphasizes the joint role of individual and institutional conditions (Rahimi, 2024), and the present study extends this view by showing how such conditions form configurations that shape externally recognized professional success.

### 6.4 Practical implications for pathway-based professional development of university English teachers

The findings suggest that universities should move from uniform teacher development policies to pathway-based professional development designs for university English teachers. A single training program or incentive policy is unlikely to serve all teachers equally well. Different groups of teachers may need different combinations of support depending on their motivational resources, institutional context, collaboration opportunities, and career stage.

First, external training support and self-efficacy development should be treated as baseline conditions. Universities should provide stable access to language teaching workshops, digital language teaching training, EAP/ESP curriculum development programs, intercultural communication training, and teaching innovation seminars. These programs should not be occasional or symbolic. They should be connected with teachers’ actual classroom practice, curriculum responsibilities, and professional recognition opportunities.

Second, academic promotion and teaching honor systems should be better aligned with professional development resources. If universities expect English teachers to pursue teaching excellence, they need to make evaluation criteria transparent and connect teaching awards with workload recognition, promotion, curriculum reform, and teaching-research output. Otherwise, honor systems may remain symbolic and fail to support sustainable professional growth.

Third, universities with stronger resource bases may adopt a resource-enrichment strategy. This means integrating external training, teaching incentives, peer collaboration, curriculum teams, and career development platforms into a coherent professional development system. Such a system is particularly suitable for universities that already have teacher development centers, teaching innovation programs, and relatively stable funding.

Fourth, universities with limited resources may adopt a resource-compensation strategy. For local or teaching-oriented institutions, where formal incentives and career development platforms may be weaker, professional development can still be supported through targeted policy support, peer mentoring, teaching-research groups, classroom observation, and time–stress management support. These lower-cost strategies may help teachers transform limited resources into visible professional achievement.

Finally, teacher development centers should use configuration-based diagnosis rather than single-indicator assessment. Instead of asking whether one resource is sufficient, administrators should identify which resource combinations are missing for different groups of English teachers. Early-career teachers may need confidence-building, mentoring, and lesson-design support; mid-career teachers may need curriculum leadership, teaching research support, and award-preparation guidance; senior teachers may need platforms for mentoring, course leadership, and external professional recognition.

### 6.5 Limitations and future research

Several limitations should be acknowledged. First, OHA was used as an institutionalized indicator of objective professional recognition, but it does not capture the full complexity of university English teachers’ professional core competence. Future studies may combine OHA with classroom observation, peer evaluation, student feedback, teaching portfolios, and longitudinal professional development records.

Second, the study used cross-sectional survey data, which limits causal inference over time. Although fsQCA can reveal configurational associations and causal complexity, it cannot fully demonstrate developmental change. Future studies may use longitudinal or panel designs to examine how university English teachers move across different resource configurations during their careers.

Third, although the measurement model showed acceptable reliability and validity, some AVE values were below the ideal threshold of 0.50. Future research may further refine the scale by adding higher-loading items and testing the model in different types of universities and language education contexts.

Fourth, the fourth configuration showed a lower consistency value than the conventional 0.80 threshold and should therefore be treated as a supplementary compensatory pattern. Future studies may examine whether this configuration remains stable in other samples, especially among local universities, vocational institutions, or universities with fewer formal teacher development resources.

Finally, this study focused on university English teachers in China. Future research may compare English teachers across different national higher education systems to examine whether the proposed configurational model is culturally specific or has broader applicability in international language teacher development.

## 7 Conclusion

This study examined how individual resources, organizational resources, and social-capital-related resources jointly shape the development of university English teachers’ professional core competencies and their attainment of objective professional recognition. By integrating Expectancy–Value Theory, the Job Demands–Resources Model, Social Capital Theory, and fsQCA, the study moves beyond previous linear understandings of teacher professional success. The findings show that high-level Objective Honor Attainment is not determined by any single factor, but is generated through multiple sufficient configurations.

The main theoretical contribution of this study lies in proposing a configurational model for explaining the development of university English teachers’ professional core competencies. This model demonstrates that professional success emerges from the synergistic interaction of motivational activation, resource alignment, and network-enabled recognition. External training support and self-efficacy–professional commitment repeatedly appear as core conditions across multiple sufficient configurations, yet neither constitutes an indispensable single prerequisite. This finding highlights the importance of equifinality, resource complementarity, and functional substitution in understanding the professional success of university English teachers.

Overall, this study argues that the professional development of university English teachers should be understood as a multi-pathway and highly context-dependent process. Teaching excellence is not achieved solely through individual competence or institutional support; rather, it results from different configurations of personal motivation, organizational resources, and professional networks. This configurational perspective enriches educational psychology research on teacher professional development and provides a theoretical basis for designing differentiated and systematic teacher development pathways in higher education.

## Data Availability

The datasets presented in this study can be found in online repositories. The names of the repository/repositories and accession number(s) can be found at: https://zenodo.org/uploads/18526229.
